# Supplementary figures and images for: Distinguishing between paediatric brain tumour types using multi-parametric magnetic resonance imaging and machine learning: A multi-site study
Source: Neuroimage Clin. 2020 Jan 23;25:102172. doi: 10.1016/j.nicl.2020.102172 (PMC7005468; doi:10.1016/j.nicl.2020.102172)

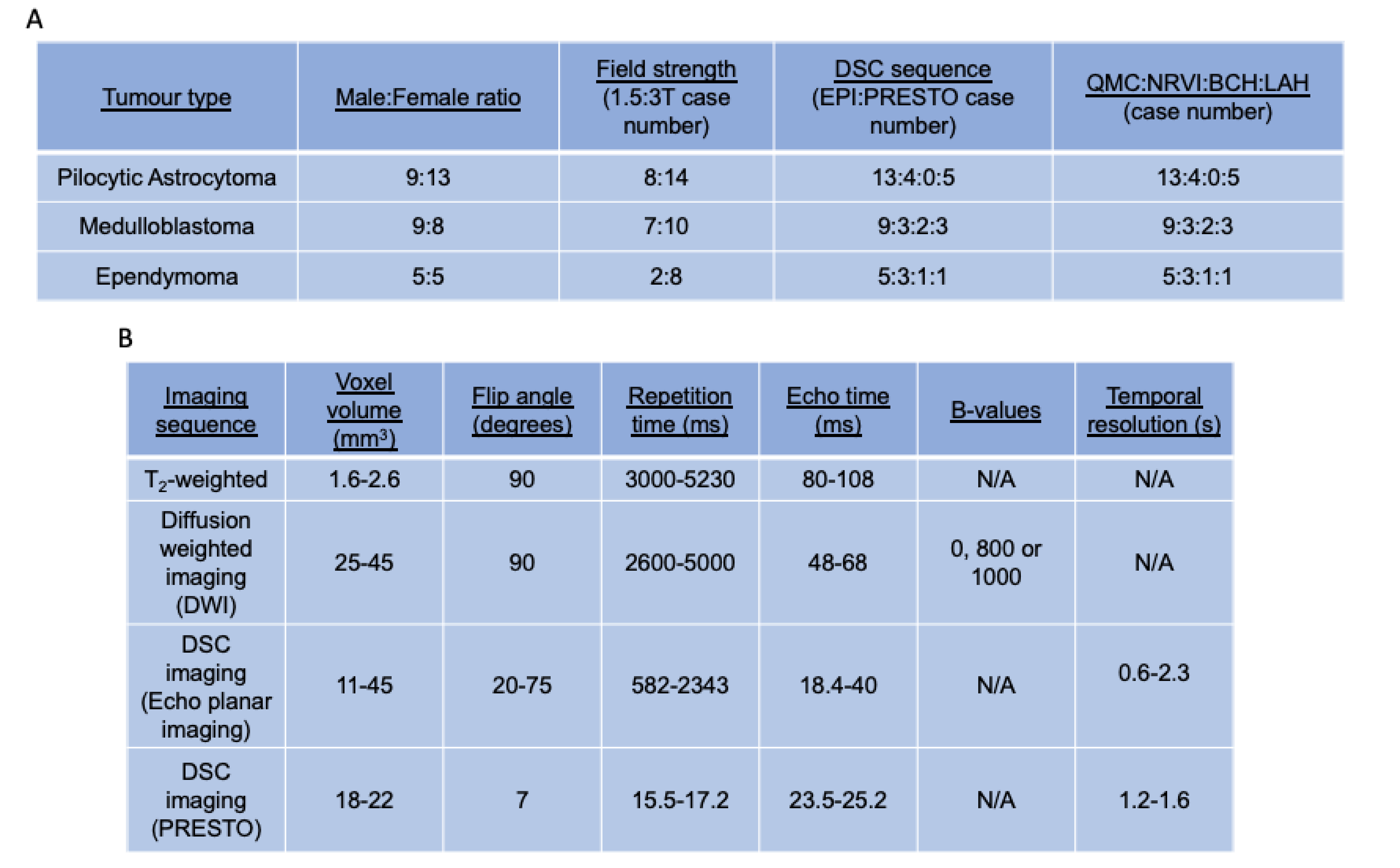

Supplement: Supplementary file 1 — Supplementary Table 1 – Cohort (A) and imaging parameters (B) used in this study. [file mmc1.jpg]

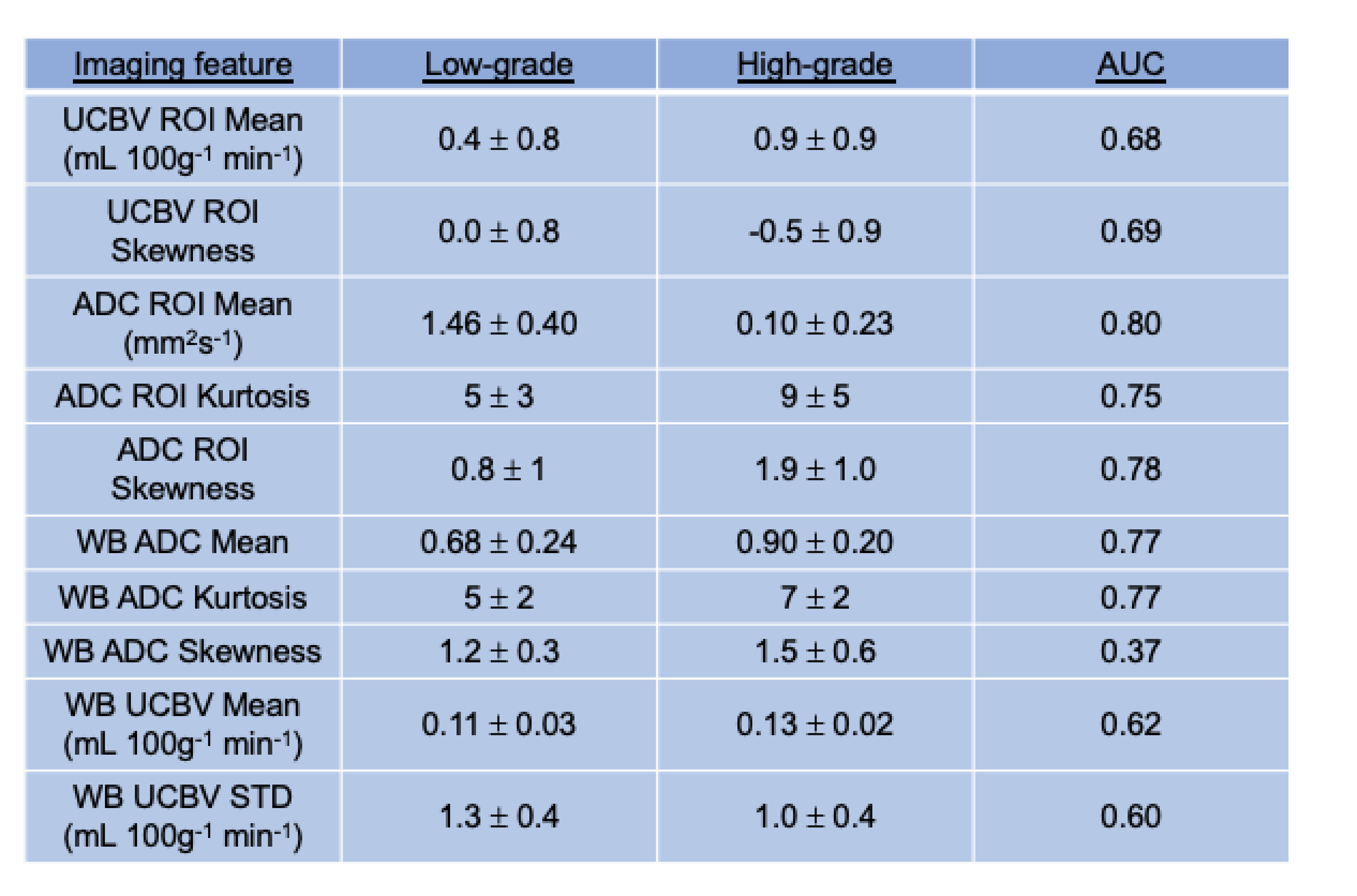

Supplement: Supplementary file 2 — Supplementary Table 2 – Significant univariate results from high/low grade separation. Analysis showed a number of significant ADC and DSC imaging features between low and high grade groups. AUC = Area under the curve. [file mmc2.jpg]

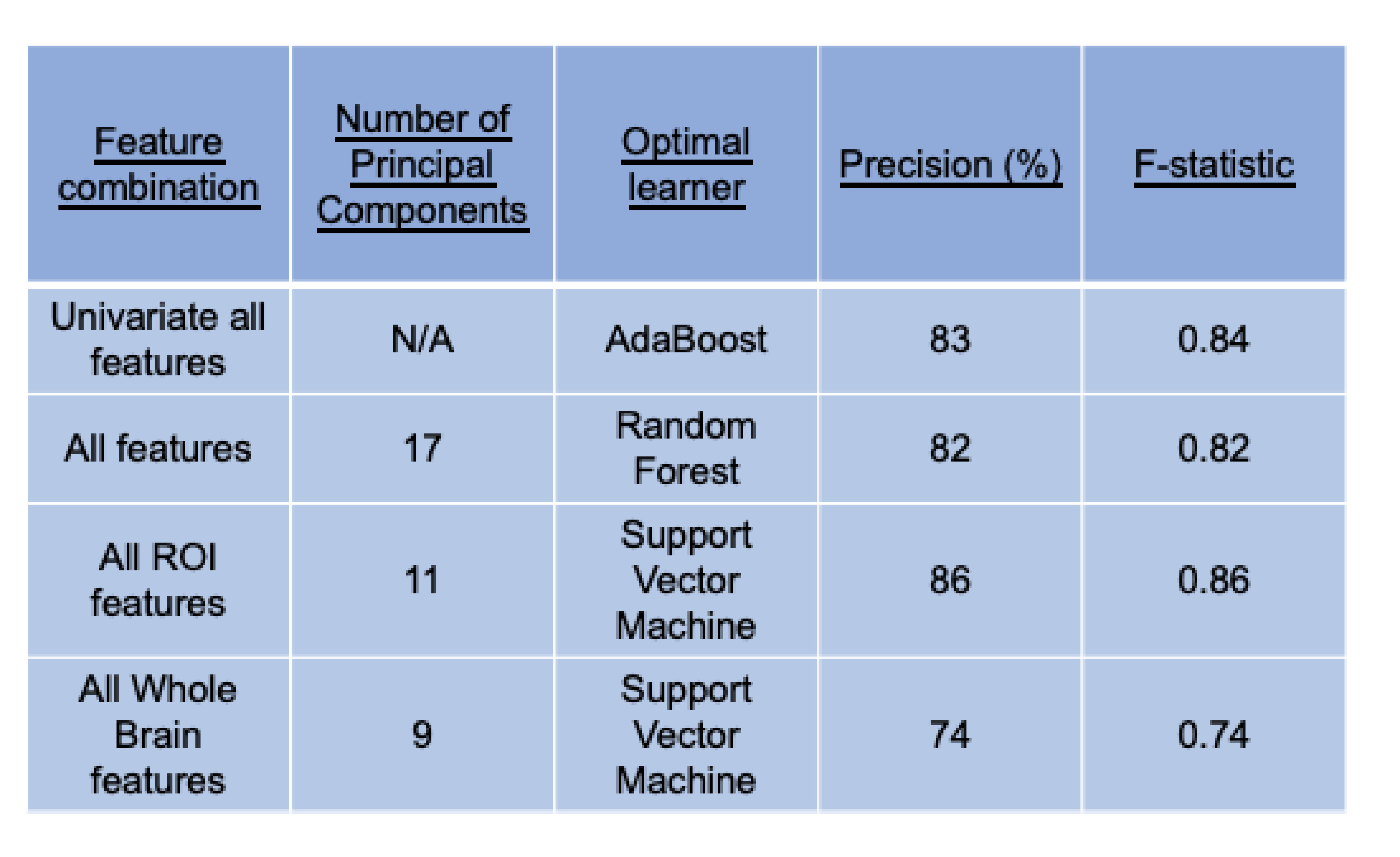

Supplement: Supplementary file 3 — Supplementary Table 3 – Supervised learning results for low/high grade. Results showed that a PCA reduced combination of ROI features combined with a Support Vector Machine provided the best learner to discriminate between high and low grade tumors. [file mmc3.jpg]

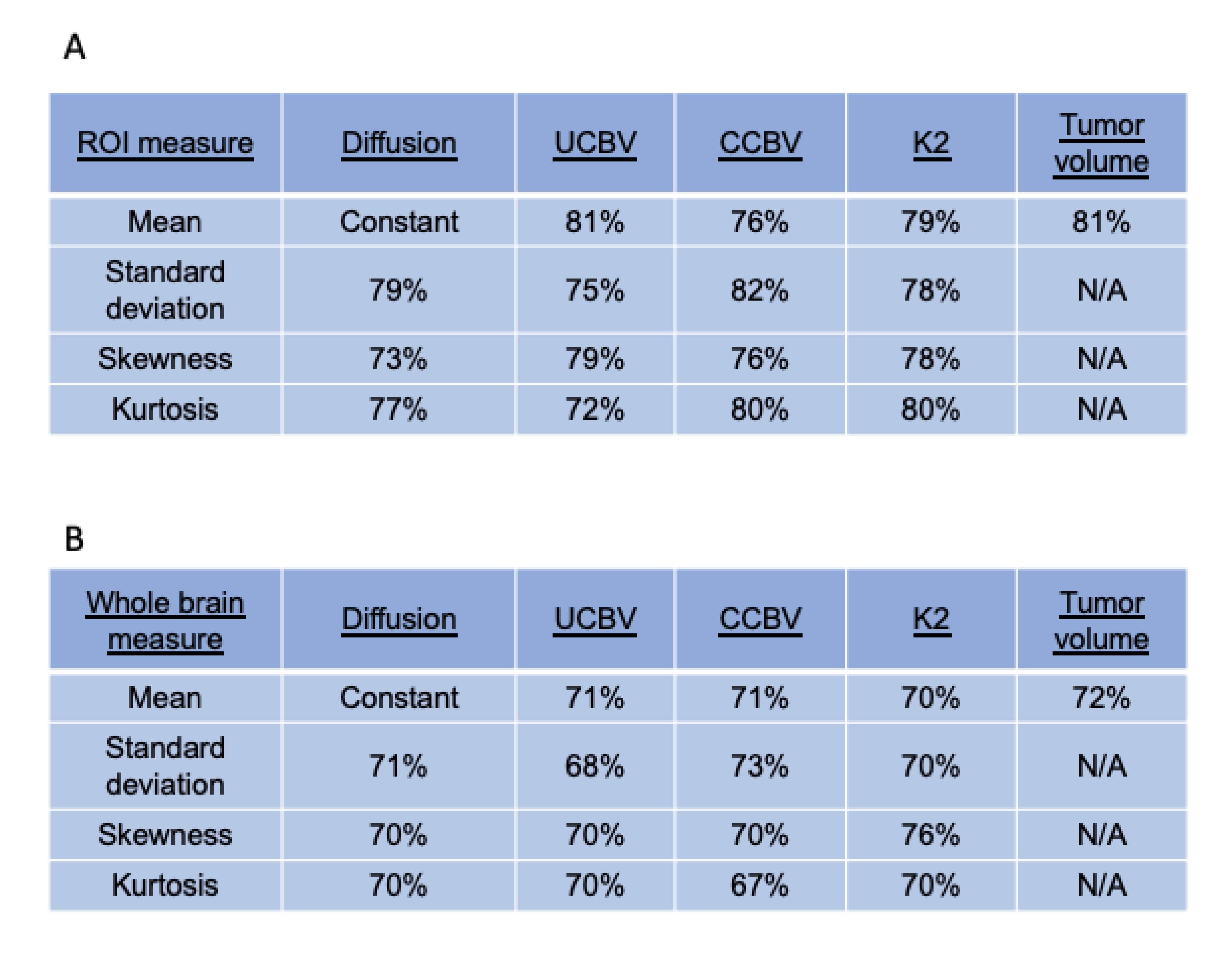

Supplement: Supplementary file 4 — Supplementary Table 4 - Perturbation testing of ROI (A) and whole brain (B) features in combination with ADC mean. [file mmc4.jpg]
